# Generation and analysis of expression sequence tags from haustoria of the wheat stripe rust fungus *Puccinia striiformis *f. sp. *Tritici*

**DOI:** 10.1186/1471-2164-10-626

**Published:** 2009-12-23

**Authors:** Chuntao Yin, Xianming Chen, Xiaojie Wang, Qingmei Han, Zhensheng Kang, Scot H Hulbert

**Affiliations:** 1 Department of Plant Pathology, Washington State University, Pullman, WA 99164-6430, USA; 2 US Department of Agricultural Research Service, Wheat Genetic, Quality, Physiology and Disease Research Unit, Pullman, WA 99164-6430, USA; 3 College of Plant Protection and Shaanxi Key Laboratory of Molecular Biology for Agriculture, Northwest A&F University, Yangling, Shaanxi, 712100, PR China

## Abstract

**Background:**

Stripe rust, caused by *Puccinia striiformis *f. sp. *tritici *(*Pst*), is one of the most destructive diseases of wheat (*Triticum aestivum *L.) worldwide. In spite of its agricultural importance, the genomics and genetics of the pathogen are poorly characterized. *Pst *transcripts from urediniospores and germinated urediniospores have been examined previously, but little is known about genes expressed during host infection. Some genes involved in virulence in other rust fungi have been found to be specifically expressed in haustoria. Therefore, the objective of this study was to generate a cDNA library to characterize genes expressed in haustoria of *Pst*.

**Results:**

A total of 5,126 EST sequences of high quality were generated from haustoria of *Pst*, from which 287 contigs and 847 singletons were derived. Approximately 10% and 26% of the 1,134 unique sequences were homologous to proteins with known functions and hypothetical proteins, respectively. The remaining 64% of the unique sequences had no significant similarities in GenBank. Fifteen genes were predicted to be proteins secreted from *Pst *haustoria. Analysis of ten genes, including six secreted protein genes, using quantitative RT-PCR revealed changes in transcript levels in different developmental and infection stages of the pathogen.

**Conclusions:**

The haustorial cDNA library was useful in identifying genes of the stripe rust fungus expressed during the infection process. From the library, we identified 15 genes encoding putative secreted proteins and six genes induced during the infection process. These genes are candidates for further studies to determine their functions in wheat-*Pst *interactions.

## Background

Rust fungi are a large group of obligately biotrophic basidiomycete fungi that completely depend on their living host tissue for growth and reproduction. Wheat (*Triticum aestivum *L.) is a host to three different rust fungi, causing stripe (yellow), leaf (brown) and stem (black) rust. Wheat stripe rust, caused by *Puccinia striiformis *Westend. f. sp. *tritici *Eriks. (*Pst*), is a serious problem in all major wheat growing countries [1,2]. In the United States, the disease is most destructive in the western United States and has become increasingly important in the south-central and south-eastern states [1,3,4]. Unlike the stem rust (*P. graminis *f. sp. *tritici*) and leaf rust (*P. triticina*) fungi, *Pst *does not have a known alternate host to complete the sexual cycle. During infection, urediniospores of *Pst *germinate on wheat leaf surfaces to produce germ tubes. Depending upon the isolate, *Pst *forms noticeable or unnoticeable appressoria [5,6], from which an infection peg is formed and penetrates a leaf stoma, followed by infection hyphae that form haustorial mother cells, and a specialized infection structure called the haustorium forms and an intimate feeding relationship is established. Haustoria are essential for rust fungi to take nutrients from their host [7-10] and have also been shown to be involved in vitamin synthesis [11].

Plant disease resistance relies on the recognition of pathogen avirulence (Avr) gene products by host resistance (R) genes through either direct (receptor-ligand model) or indirect (guard model) association, which induces defense responses. Haustoria play an essential role in the reactions of plants with rust fungi. For example, four avirulence genes from *Melampsora lini*, the flax rust pathogen, have been cloned and found to encode small secreted proteins expressed in the fungal haustoria [12,13]. A large number of plant-induced and haustorium-specific genes have been identified in the bean rust fungus *Uromyces fabae *[14,15]. To date, there are no reports of cloning and molecular characterization of either virulence or avirulence genes from any of the cereal rust pathogens.

The stripe rust fungus lacks several features to be an ideal model system for genetic analysis. It does not have a known alternate host for completing the sexual cycle. Like the other cereal rusts, *Pst *is very difficult to culture in vitro and stable transformation systems are yet to be developed. While molecular and genetic approaches are currently lacking, some advances are being made in genomics. Recently Ling et al. [16] constructed a full-length cDNA library of *Pst *from RNA extracted from urediniospores and identified some genes encoding protein products that maybe involved in virulence or infection. Some genes highly expressed in germinated urediniospores of the fungus were also reported [17]. Our understanding of the molecular mechanisms underlying infection and development within host tissue is still very limited. A haustorium is a hub of cellular communication between the host and the pathogen for the establishment of a biotrophic relationship. To gain some insights into haustorium-related functions and investigate *Pst *virulence mechanisms, we constructed a *Pst *haustorial cDNA library based on a protocol for the preparative isolation of haustoria from rust-infected leaves [18] and searched for expressed sequence tags (ESTs) encoding putative secreted proteins. More than 5,000 ESTs from the haustorial cDNA library were generated. Fifteen unique sequences were predicted to encode proteins secreted from haustoria. Quantitative real-time PCR (qRT-PCR) studies revealed that some cDNAs were specifically expressed *in planta*.

## Results

### Construction of a haustorial cDNA library

Stripe rust haustoria were isolated from heavily infected wheat leaves. Total RNA was extracted from haustoria of race PST-78 of *Pst *and a cDNA library was constructed with the pDNR-LIB vector. Most of the cloned cDNA inserts in this library were between 300-1,500 bp in size. A total of 6,000 random cDNA clones were sequenced from the 5' end, from which 5,126 high quality ESTs were obtained. While the sequencing reactions covered the full inserts of many of the smaller clones, 687 of the clones with larger inserts were also sequenced from the 3' end.

### EST sequence analysis

**Figure 1:**
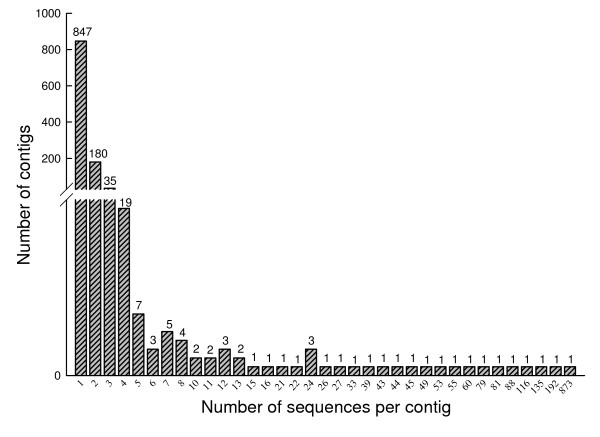
Frequency distribution of sequences from the haustorial cDNA library of *Puccinia striiformis *f. sp. *tritici *belonging to the same contig or unisequence. A total of 1,134 unisequences were used in this analysis

The EST sequences were subjected to BLAST searches (described in methods). Of the 5,126 sequences, 1,420 sequences were found to be likely of plant origin as indicated by significant BLAST scores (E value ≤ 10^-5^) to plant sequences but little or no homology to other organisms. After removing contaminating plant sequences, 3,706 sequences were assembled into 1,134 unique sequences, of which 847 were singletons and 287 were contigs represented by multiple clones at frequencies ranging from 2 to 873. A majority of the contigs contained two, three or four sequences. The frequency of redundant ESTs was shown in Figure 1. The average G+C content of these unique sequences was 43.11%, which was similar to the G+C content of ESTs in *P. graminis *and *Pst *germinated urediniospores [17,19]. The sequences were deposited in the NCBI dbEST sequence database (Accession numbers GH737012 - GH738498).

**Figure 2:**
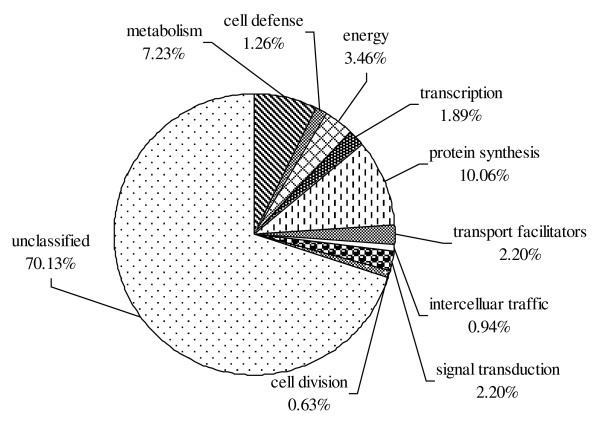
Functional classification of the *Puccinia striiformis *f. sp. *tritici *unisequence from the haustorial cDNA library showing significant similarities to proteins in public databases

All unique sequences were used in homology searches of the NCBI non-redundant protein sequences and the *P. graminis *genome database using the BLASTX and BLASTn algorithm. Unique sequences with significant homology (E ≤ 10^-5^) to known proteins were grouped according to their putative functions [see additional files 1 and 2]. Of the 1,134 unique sequences, only 109 (10%) showed significant similarities to proteins of known function, 296 (26%) showed significant similarities to predicted proteins of unknown function and 729 (64%) showed no significant similarity to a database entry. Based on the examination of the significant sequence similarity to a database entry, a putative functional category was assigned to the specific unisequence. The majority of the genes were predicted to code for proteins of unknown function (Figure 2). The largest group of genes with known functions showed similarities to ribosomal proteins, followed by the group of genes with similarities to proteins involved in primary metabolism and energy production. Some unisequences matched ESTs from *Pst *urediniospores [16] and germinated urediniospores [17] deposited in the Genbank. Some had high homology to ESTs from a haustorium-specific cDNA library of *U. fabae *[15].

### Prediction of ESTs encoding secreted proteins

To identify putative secreted proteins from the haustorial cDNA library, we selected unisequences that appeared to code for full-length open reading frames and predicted their translation products. Predicted proteins from open reading frames with in-frame stop codons before the start codon were analyzed with the signal P 3.0 algorithm [20] and iPSORT [21]. The analysis identified 15 unisequences encoding proteins with secretion signal peptides at the N-terminus (Table 1). These sequences were predicted to encode proteins ranging in size from 56-289 amino acids. Seven of them did not show significant homology to known protein sequences, seven matched predicted proteins of unknown function and one had significant homology to sulfate transporters. Eight encoded Cys-rich proteins with more than 5% cystein residues in the predicted protein.

**Table 1:** Genes from the haustorial cDNA library of *Puccinia striiformis *f. sp. *tritici *predicted to encode secreted proteins

Unisequence	GenBank accession	Size (aa)	No. of Cysteine residues	Homology in databases	E value
PSTha2a5	GH737102	117	6	predicted protein of *Puccinia graminis*	5.23E-23
PSTha9F18	GH737274	259	14	hypothetical protein of *Puccinia graminis*	8.96E-41
PSTha12a4	GH737444	289	10	predicted protein of *Puccinia graminis*	4.00E-23
PSTha12j12	GH737467	133	6	no homology	-
PSTha15N21	GH737567	98	7	no homology	-
PSTha21O8	GH737139	92	1	putative sulfate transporter	1.42E-38
PSTha5a23	GH737046	108	4	no homology	-
PSTha6i16	GH737950	73	5	predicted protein of *Puccinia graminis*	3.00E-10
PSTha8F13	GH738007	116	6	no homology	-
PSTha2c7	GH737231	204	13	predicted protein of *Puccinia graminis*	1.15E-15
PSTha16B3	GH737129	87	2	no homology	-
PSTha10F24	GH737323	56	3	no homology	-
PSTha16D6	GH737598	66	3	hypothetical protein of *Aspergillus niger*	2.00E-16
PSTha9C13	GH738022	65	1	predicted protein of *Puccinia graminis*	6.00E-10
PSTha12h2	GH737173	70	5	no homology	-

### Expression patterns of genes from haustorial cDNA library

To examine developmental stage-specific gene expression, ten unisequences were selected from the haustorial cDNA library to assay their transcript levels during different developmental and infection stages through qRT-PCR. As determined by the GeNorm analysis, elongation factor-1, β-tubulin and actin showed M values of 0.699, 0.779 and 1.838. Elongation factor-1 was the most stable gene and was therefore used as a reference to normalize gene expression across different samples. Gene expression of the ten genes in uninfected wheat leaves, urediniospores, *in vitro *germinated urediniospores and infected wheat leaves was shown in Table 2. Among these genes, six were putative secreted proteins (PSTha2a5, PSTha12j12, PSTha5a23, PSTha12a4, PSTha12h2 and PSTha9F18). PSTha5a23 was specifically expressed in plants and was either not expressed in urediniospores and germinated urediniospores or expressed very weekly. Expression levels of PSTha2a5 and PSTha12j12 were weak in urediniospores, slightly increased in germinated urediniospores and high in *Pst*-infected leaves. PSTha12h2 was expressed in all stages of development, with the highest levels in infected leaves. PSTha12a4 transcripts only increased in germinated urediniospores but not in infected leaves. In contrast, PSTha9F18 was expressed strongly in urediniospores and germinated urediniospores, and its expression decreased dramatically in infected leaves.

**Table 2:** Expression of *P. striiformis *f. sp. *tritici *genes in different developmental stages

Unisequence	GenBank accession	Relative expression		
		
		**GerUred/Ured**^a^	InfW/GerUred^b^	InfW/Ured^c^
PSTha2a5	GH737102	4.70 ± 1.19	25.81 ± 9.88	119.89 ± 48.27
PSTha12j12	GH737467	9.40 ± 3.03	11.40 ± 3.06	104.31 ± 28.23
PSTha14i19	GH737527	0.55 ± 0.10	350.07 ± 56.80	192.38 ± 41.06
PSTha5a23	GH737046	3.15 ± 0.70	1165.64 ± 229.00	3617.32 ± 607.41
PSTha5a1	GH737242	3.09 ± 0.54	1948.90 ± 238.26	5980.69 ± 867.12
PSTha12a4	GH737444	5.70 ± 2.87	0.71 ± 0.31	3.68 ± 1.22
PSTha12h2	GH737173	0.47 ± 0.05	50.40 ± 5.14	23.48 ± 3.10
PSTha 9F18	GH737274	1.42 ± 0.34	8.11E-04 ± 1.42E-04	1.14E-03 ± 3.08E-04
PSTha5G19	GH737890	1.42 ± 0.82	0.2 ± 0.11	0.26 ± 0.16
PSTha11n16	GH737421	0.94 ± 0.22	0.22 ± 0.06	0.20 ± 0.05

^a ^ratio of expression in germinated urediniospores (GerUred) vs. expression in urediniospores (Ured)

^b ^ratio of expression in infected leaves (InfW) vs. expression in germinated urediniospores (GerUred)

^c ^ratio of expression in infected leaves (InfW) vs. expression in urediniospores (Ured)

The other four genes for which expression patterns were examined were a *THI2P *homolog (PSTha14i19), a calcium/calmodulin-dependent protein kinase 2 homolog (PSTha11n16), a predicted chitinase (PSTha5a1) and a ubiquitin ligase E3C homolog (PSTha5G19). PSTha14i19 was expressed with the highest level in infected leaves. PSTha14i19 was homologous to *U. fabae *PIG4, which was thought to be involved in vitamin B1 biosynthesis [11,14,15]. A similar *in planta *induced rust gene was also reported in *P. triticina*, a wheat leaf rust pathogen [22-24]. Transcripts for PSTha5a1 were detected only in plants, while PSTha5G19 and PSTha11n16 were down regulated in infected leaves. None of these genes had transcripts in uninfected wheat leaves (data not shown), indicating that they were indeed *Pst *genes.

## Discussion

Haustoria are specialized structures that are formed within the living cell of a host by biotrophic fungal pathogens during infection. Previous analyses of haustorial transcripts from other rust fungi [13-15,25] indicated that they are rich in sequences induced *in planta *and involved in virulence as well as other aspects of parasitism, like nutrient uptake. In the present study, we constructed a cDNA library from the wheat pathogen *P. striiformis *f. sp. *tritici *haustoria. A total of 5,126 randomly chosen ESTs were generated which represented 1,134 unique transcripts once plant sequences were removed and redundancies eliminated. Most of the highly redundant sequences, like several coding for ribosomal proteins, were very similar to other fungal sequences in databases and/or sequences found in *Pst *urediniospore and germinated urediniospore cDNA libraries and a *U. fabae *haustorium-specific cDNA library [15-17]. Proteins involved in protein synthesis, primary metabolism and energy production were most prevalent in predicted proteins with known functions. Similar findings were also reported in other pathogenic interactions during infection [19,22,24]. Overall, the less redundant sequences were surprisingly unique, especially considering the database searches included nucleotide and predicted protein searches of the *P. graminis *f. sp. *tritici *genome sequence and nucleotide searches of the *Melampsora larici-populina *genome sequence. Approximately 64% of the 1,134 unique sequences did not show significant similarities to known genes in databases. The frequency of sequences with no matches would likely have been lower if the full sequence of the cDNAs were available; some sequences appeared mostly non-coding. However, this frequency was not only high compared to other fungi [15,26] but also higher than that observed for *Pst *libraries made from urediniospores [16] or germinated urediniospores [17]. Avirulence proteins from eukaryotic plant pathogens described to date have indicated that they are diverse in function and have "novel" sequences with little sequence similarity to proteins of known function. If genes expressed in haustoria are more likely involved in virulence, which is probably more specific to particular fungal species, this might explain why a high percentage of genes expressed in haustoria share little or no sequence similarity to genes in other fungi.

Several of the haustorial transcripts were predicted to encode products homologous to potential virulence-related proteins based on sequence comparison with other plant or animal pathogens. For example, PSTha15i2 was homologous to glutamine synthetase. Several studies have indicated that glutamine metabolism is important for the virulence of various pathogens [27-29]. Glutamine synthetase enzyme activities were detected in pathogenic species of *Mycobacterium*, but were not detected in non-pathogenic species, indicating that this activity is potentially involved in the pathogenicity [30]. The glutamine synthetase of *U. fabae *was more strongly expressed *in planta *than in germinated urediniospores [15]. PSTha15B19 is a putative *lpd *gene encoding dihydrolipoyl dehydrogenase. The mutant of the *lpd *gene in *Mycoplasma gallisepticum *resulted in reduced virulence [31].

Several unisequences were candidates for genes encoding the key components of conserved signaling pathways. PSTha18c1, PSTha10L9 and PSTha12p13 were found to encode cAMP-dependent protein kinase type 2, protein ras-1 precursor and protein phosphatase PP2A regulatory subunit B, respectively. cAMP-dependent protein kinase pathway elements are remarkably conserved and effects on virulence have been the focus of many studies. In *Cryptococcus neoformans*, the cAMP signaling cascade is required for both melanin and capsule production, and mating filaments. All elements of the cAMP cascade are essential for the serum-induced switch of yeast to hyphal growth, which is important for the virulence of this fungus [32]. The cAMP cascade regulates pathogenicity of *Ustilago maydis*[33]. RAS proteins belong to the Rho family (a superfamily of GTPases). The *ras1 *mutants of *C. neoformans *were avirulent in animals [34]. Protein phosphatase PP2A is involved in several signal transduction pathways. Disruption of *rgb1 *gene, a subunit of PP2A, in *Sclerotinia sclerotiorum *reduced pathogenesis [35]. Further investigations of these proteins in *Pst *haustoria would be necessary to elucidate their functions in the infection process.

The fifteen unigenes predicted to encode secreted proteins are likely the best candidates for genes involved in specific virulence. Many plant pathogens manipulate their hosts through delivery of effector proteins [36-39]. In contrast to host resistant proteins, rust and mildew avirulence gene products described to date often share no significant sequence similarity to proteins of known function [36]. About 30 *Avr *genes in the flax rust pathogen have been identified by genetic analysis [40]. Recently Ellis and coworkers identified 21 HESPS (haustorially expressed secreted proteins) genes by examining a *M. lini *haustorium transcripts for secretion signals [12]. Among these HESPS, three co-segregated with the independent *AvrM*, *AvrP4 *and *AvrP123 *loci. Transient expression assays have shown that these genes function as avirulence determinants to induce R gene-dependent cell death in flax [12]. This indicates that avirulence proteins are very abundant among proteins secreted from haustoria and thus the haustorial libraries are very useful tools for identifying them. In the present study, we identified 15 putative secreted proteins from haustoria. The sequences of these proteins provided few clues to their functions except for PSTha21O8, which had significant sequence similarity to a sulfate transporter. Understanding the functions of these genes should shed light on the mechanisms of *Pst *virulence and biotrophism. Ongoing efforts to establish methods for stable transformation and transient expression assays in this biotrophic fungus are therefore a high priority.

## Conclusions

A cDNA library was constructed from RNA of haustoria isolated from *Pst*-infected wheat leaves. A total of 5,126 EST sequences of high quality were generated and assembled into 1,134 unique sequences. Approximately 64% of them showed no significant similarities in public databases, indicating that many are likely specific to certain *Puccinia *taxa and valuable for future genomic studies of the stripe rust pathogen. Most of the transcripts with known functions were predicted to encode ribosomal proteins involved in protein synthesis, followed by proteins of primary metabolism and energy production. Some of the unisequences were predicted to encode products that exhibited high similarities to proteins potentially associated with virulence from other fungi. The 15 haustorium-specific genes predicted to encode secreted proteins are candidates for future studies to determine their potential functions in the wheat-*Pst *interactions.

## Methods

### Plant genotypes and *Pst *isolates

A wheat line carrying the *Yr8 *resistance gene in the 'Avocet Susceptible' background was inoculated with race PST-78 for production of spores and haustoria. Inoculation and culturing the host and pathogen was performed as described by Chen and Line [41]. To extract RNA from urediniospores, germinated urediniospores and infected wheat leaves, fresh urediniospores were harvested from infected leaves 15 days post inoculation (dpi). Urediniospores were germinated as previously described [17]. Briefly, fresh urediniospores were suspended in sterile distilled water in glass petri dishes and incubated in the dark for 12-15 h at 10°C. Infected wheat leaves were harvested at 8 dpi with PST-78. Fresh urediniospores, germinated urediniospores, uninfected leaves and infected leaves were frozen in liquid nitrogen, and stored at -80°C for further use.

### Isolation of *Pst *haustoria

Haustoria were isolated from heavily infected wheat leaves at 8 dpi (just prior to sporulation) using ConA affinity chromatography as described by Hahn and Mendgen [18]. Twenty-five g of infected wheat leaves were gently washed with chilled distilled water and homogenized in 160 ml of homogenization buffer [0.3 M sorbitol, 20 mM MOPS pH7.2, 0.1% BSA, 0.2% 2-mercaptoethanol, 0.2% PEG 6000] using a blender at maximum speed for 10 s. The homogenate was passed through a 20 μm nylon mesh and centrifuged at 5,000 g for 5 min. The resulting pellet was resuspended in 8 mL of suspension buffer. The suspension was centrifuged at 5,000 g for another 5 min. The pellet was resuspended in 4 mL of suspension buffer. Two 2 mL aliquots of the suspension were loaded onto two columns each filled with 4-5 mL of a sepharose 6MB coupled to ConA. The columns were incubated for 15 min after the aliquots entered the columns. After extensive washing of the columns with suspension buffer, haustoria were released from the columns by agitation using a wide-bore sterile pipette. The binding and washing steps were repeated 3-4 times in fresh columns, until most of the chloroplasts had been washed away. The haustoria in the suspension buffer were then transferred to a 15 mL Eppendorf tube and centrifuged for 1 min at 10,000 g and the pellet frozen in liquid nitrogen and stored at -80°C. The whole process was carried out with reagents at 4°C.

### Isolation of RNA

For RNA isolation, fresh urediniospores, germinated urediniospores, infected leaves, uninfected leaves and haustorial cells were ground separately in a mortar in liquid nitrogen. Total RNA was isolated from frozen powder using the Qiagen Plant RNeasy kit (Qiagen, Chatsworth, GA) according to the manufacturer's instruction. For qRT-PCR analysis, RNA samples were treated with DNase I and purified with phenol/chloroform. The absence of genomic DNA contamination was subsequently confirmed by the null PCR amplification of RNA samples with primers designed for the *Pst *βtubulin gene and wheat GAPDH gene. The quantity and purity of isolated total RNA was analyzed by 2% agarose gel electrophoresis as well as by using a spectrophotometer.

### Construction and sequencing of a haustorial cDNA library

A cDNA library was prepared from 1 μg of total RNA from haustoria using the SMART™ cDNA library construction kit (Clontech, USA) according to the manufacturer's instruction. The colonies were subsequently picked and arrayed into 384-well micro-titer plates. Each well on the culture plate contained 75 μL of LB freezing storage medium [360 mM K_2_HPO_4_, 132 mM KH_2_PO_4_, 17 mM Na citrate, 4 mM MgSO_4_, 68 mM (NH_4_)_2_SO_4_, 44% (v/v) glycerol, 12.5 μg/ml of chloramphenicol, LB]. Colonies were incubated at 37°C overnight, and then stored at -80°C. Prior to sequencing, 52 clones were randomly picked to check for the presence of inserts by colony-PCR using the M13 forward and reverse primers. cDNA clones were sequenced with primer seq1 (5' CGACTCTAGACTCGAGCAAG 3') from the 5'-end. Some clones with larger insert sizes were also sequenced with primer seq2 (5' AACAGCTATGACCATG 3') from the 3'-end using an ABI 3130-XL DNA sequencer.

### Sequence analysis and database searches

Raw sequences were processed using cross-match [42], which resulted in the removal of poor quality sequences and vector sequences. Assembly of individual sequences into overlapping contigs was done as described [17]. Contaminating wheat sequences were removed from sequences by BLASTn of the NCBI non-redundant database and the 'dbEST_Others' (non-mouse, non-human) and BLASTX of the NCBI non-redundant protein sequences. The remaining ESTs were further compared with the NCBI non-redundant protein database and *P. graminis *f. sp. *tritici *genomic database using the BLASTX and BLASTn program http://www.broadinstitute.org/annotation/genome/puccinia_graminis/Blast.html and *Melampsora larici-populina *genomic database using the BLASTn program http://genomeportal.jgi-psf.org/cgi-bin/runAlignment. E-values of less than 10^-5 ^were considered significant matches to database sequences.

### Secreted protein prediction

To search for potential secreted proteins, the sequences from the haustorial cDNA library were analyzed with the SignalP 3.0 algorithm [[20], http://www.cbs.dtu.dk/services/SignalP/]. To support the SignalP result, the protein sequences with predicted secretory signal peptides were also examined using iPSORT http://hc.ims.u-tokyo.ac.jp/iPSORT/.

### Quantitative real-time PCR analysis

Using 1 μg of RNA isolated from fresh uninfected leaves, urediniospores, germinated urediniospores and infected leaves, reverse transcriptions were performed using superscript reverse transcriptase with an oligo (dT15) primer (Promega. Madison, WI. USA) according to the manufacturer's instructions. qRT-PCR was carried out using the Bio-Rad iQ5 Real-Time PCR system. Specific primers for each gene selected were designed using primer design software (Integrated DNA Technologies) and listed in Table 3. Real Time PCR was conducted in 20 μL volumes using SYBR Green PCR master mix (sigma). PCR conditions used were 95°C for 15 min, followed by 50 cycles of 95°C for 20 s, 55-60°C for 30 s and 72°C for 30 s, followed by a melting curve program. To identify a housekeeping gene with minimal variability in different rust developmental and infection stages, a qRT-PCR assay was established for three candidate reference genes (βtubulin, elongation factor-1 and actin) to analyze their transcription levels. Subsequently, the reference gene stability measures (M) were calculated by using the GeNorm tool as previously described [43]. A minimum of three biologically independent samples were used for each developmental stage, and two technical replicates were performed on every sample. Standard curves were generated for each gene. The quantification of gene expression was performed using the relative standard curve method by comparing the data with the reference gene.

**Table 3:** Primers used in qRT-PCR analysis to determine expression patterns of ESTs from the *Puccinia striiformis *f. sp. *tritici *haustorial library in various developmental and infection stages

Primer	Sequence(5' to 3')	Products size (bp)
RT-PSTha2a5-F	TGAATGGGTCGGTTGCCACAGATA	180
RT-PSTha2a5-R	GGCCCAAAGGGAATGGTCGAATTT	
RT-PSTha12j12-F	GCTTCGTTCGGGATTCAAAGCAAC	138
RT-PSTha12j12-R	ACATCTTGGGAACAGGCAGTTTCG	
RT-PSTha14i19-F	AAGTGCTCGAATGGGTCCTACCTT	135
RT-PSTha14i19-R	TGTGACGTTCACTTAGCCGATCCA	
RT-PSTha12h2-F	ACGTCAGTCAAAGATGTCGGCGAA	120
RT-PSTha12h2-R	TTCCTATCAATTAGCGCGGGAGCA	
RT-PSTha5a23-F	TTCCTACTCTGGCGACCAACATCA	194
RT-PSTha5a23-R	AAATCCGACTGACCGACATCCGTT	
RT-PSTha5a1-F	ACCGTATCGAAAGTGGTGTACGCT	82
RT-PSTha5a1-R	TGTCGTCCATTGGTCCCATAGTGT	
RT-PSTha12a4-F	GTTCACCAAAGCCACCTTCAACCA	128
RT-PSTha12a4-R	ATTAGACGGCGGCGTTCTTAGGAT	
RT-PSTha9F18-F	ATTCGAGATTAACGCGACCAACGG	169
RT-PSTha9F18-R	GAAAGGTCAATGACAACGGCGTCT	
RT-PSTha5G19-F	AGGTCTCGATTACCTTCCGCTTCT	127
RT-PSTha5G19-R	AAGAAAGATCGAAACCAGCACCAG	
RT-PSTha11n16-F	TGGGCATCTTCAGCTAGTTGGACT	181
RT-PSTha11n16-R	TCAACACATTCAGACCACCTCCGA	
RT-EF1-F	TTCGCCGTCCGTGATATGAGACAA	159
RT-EF1-R	ATGCGTATCATGGTGGTGGAGTGA	
RT-GAPDH-F	CAACGCTAGCTGCACCACTA	161
RT-GAPDH-R	TTCCACCTCTCCAGTCCTTG	
RT-TUB-F	CCGATCAATTCACGGCCATGTTCA	174
RT-TUB-R	AACCCTCTTCAACTTCCTCGTCGT	
RT-ACT-F	TGTCGGGTGGAACGACCATGTATT	146
RT-ACT-R	AGCCAAGATAGAACCACCGATCCA	

## Authors' contributions

CY isolated haustoria, constructed the haustorial cDNA library, participated in EST sequence analysis, conducted qRT-PCR and drafted the manuscript; XC contributed materials and resources, and wrote and revised the manuscript; XW, QH and ZK contributed to EST sequencing and BLAST searches. SH conceived and coordinated the study, interpreted the data, and wrote and revised the manuscript. All authors read and approved the final manuscript.

## Supplementary Material

Additional file 1 Putative functions of expressed sequence tags from the haustorial cDNA library of *Puccinia striiformis *f. sp. *tritici *as determined by BLASTX searches of the NCBI database Click here for file

Additional file 2 Putative functions of expressed sequence tags from the haustorial cDNA library of *Puccinia striiformis *f. sp. *tritici *as determined by BLASTX searches of the *P. graminis *genome database Click here for file
